# Clinician agreement and influence of medication‐related characteristics on assessment of polypharmacy

**DOI:** 10.1002/prp2.321

**Published:** 2017-05-31

**Authors:** Gao‐Jing Ong, Amy Page, Gillian Caughey, Sally Johns, Emily Reeve, Sepehr Shakib

**Affiliations:** ^1^ Department of Clinical PharmacologyRoyal Adelaide HospitalNorth TerraceAdelaideSouth AustraliaAustralia; ^2^ School of Medicine and PharmacologyUniversity of Western AustraliaPerthAustralia; ^3^ School of Pharmacy and Medical SciencesUniversity of South AustraliaAdelaideSouth AustraliaAustralia; ^4^ Cognitive Decline Partnership CentreKolling Institute of Medical ResearchSydney Medical SchoolThe University of SydneyNew South WalesAustralia; ^5^ Geriatric Medicine Research UnitDalhousie University and Nova Scotia Health Authority5955 Veterans' Memorial LaneHalifaxNova ScotiaCanada; ^6^ Discipline of PharmacologySchool of MedicineUniversity of AdelaideNorth TerraceAdelaideSouth AustraliaAustralia

**Keywords:** Deprescribing, inappropriate prescribing, older adults, polypharmacy

## Abstract

It is not known how clinicians assess polypharmacy or the medication‐related characteristics that influence their assessment. The aim of this study was to examine the level of agreement between clinicians when assessing polypharmacy and to identify medication‐related characteristics that influence their assessment. Twenty cases of patients with varying levels of comorbidity and polypharmacy were used to examine clinician assessment of polypharmacy. Medicine‐related factors within the cases included Beers and STOPP Criteria medicines, falls‐risk medicines, drug burden index (DBI) medicines, medicines causing postural hypotension, and pharmacokinetic drug–drug interactions. Clinicians were asked to rate cases on the degree of polypharmacy, likelihood of harm, and potential for the medication list to be simplified. Inter‐rater reliability analysis, correlations, and multivariate logistic regression analyses were conducted to identify medicine factors associated with clinicians' assessment. Eighteen expert clinicians were recruited (69.2% response rate). Strong agreement was observed in clinicians' assessment of polypharmacy (intraclass correlation coefficients [ICC] = 0.94), likelihood to cause harm (ICC = 0.89), and ability to simplify medication list (ICC = 0.90). Multivariate analyses demonstrated number of medicines (*P* < 0.0001) and DBI scores (*P* = 0.047) were significantly associated with assessment of polypharmacy. Medicines associated with harm were significantly associated with the number of medicines (*P* = 0.01) and Beers criteria medicines (*P* = 0.003). Ability to simplify the medication regimen was significantly associated with number of medicines (*P* = 0.03) and medicines from the STOPP criteria (*P* = 0.018). Among clinicians, strong consensus exists with regard to assessment of polypharmacy, medication harm, and ability to simplify medications. Definitions of polypharmacy need to take into account not only the numbers of medicines but also potential for medicines to cause harm or be inappropriate, and validate them against clinical outcomes.

**Abbreviations**
ICC: intraclass correlation coefficients
DBI: drug burden index
MACS: multidisciplinary ambulatory consultation service

## Introduction

The presence of multiple chronic conditions (multimorbidity) is common in the older population (Wallace et al. 2015). As a consequence, multiple medicine use or polypharmacy is also common in the older population, to control symptoms, prevent disease complications, and prevent development of new medical conditions (Wallace et al. 2015). A recent population‐based UK study reported that 40% of those aged 65 years and older are dispensed five or more medications, with 17.2% dispensed 10 or more medications (Guthrie et al. 2015). As the population worldwide continues to age, the prevalence of polypharmacy and the average number of medications that older adults take each day is increasing (Hovstadius et al. 2010; Guthrie et al. 2015). While these medications have the potential to achieve substantial benefits, they can also result in substantial harms (Hilmer and Gnjidic 2009). Polypharmacy is independently associated with poor clinical outcomes in older adults, including falls, frailty, impaired cognition, increased hospital admissions, and adverse drug reactions (Hajjar et al. 2007; Hilmer and Gnjidic 2009; Gnjidic et al. 2012). Increasing numbers of medications is associated with increasing risk of adverse events; the likelihood of an older person having an adverse drug event increases from 10% if one medication is taken to 75% if five or more medications are used (Byles et al. 2003).

Polypharmacy is strongly associated with increased use of potentially inappropriate medicines (Steinman et al. 2006). An inappropriate medication is one where the potential harms of the medication use outweigh the potential benefits, or where the use of the medication does not align with the individual's preferences and/or goals of care. Explicit lists of medications which are considered to be potentially inappropriate in older adults due to their high risk of harm in this population, such as the Beers and STOPP/START criteria have been developed (O'Mahony et al. 2015; The American Geriatrics Society Beers Criteria Update Expert Panel, 2015) and use of potentially inappropriate medicines has been shown to be independently associated with medication‐related harms (Cahir et al. 2014).

Polypharmacy in older adults may be paradoxically associated with undertreatment (Kuijpers et al. 2008; Cherubini et al. 2012). That is, despite the number of medications taken, older adults are less likely to be prescribed one or more medicines indicated for the conditions present, potentially reducing quality of life and survival benefits (Kuijpers et al. 2008; Cherubini et al. 2012). This highlights the need to assess the overall quality and appropriateness of the medication regime and not just the count of medications taken.

Polypharmacy has been defined in many ways. Concurrent regular use of five or more medications is the most commonly used, but definitions between two and ten concurrent medications have been used (Turner et al. 2015). However, the number of medications may not be the best indicator of prescribing quality in a clinical setting and differentiation between appropriate and inappropriate polypharmacy may be more relevant (Belfrage et al. 2015; Garfinkel and Bahat 2015; Scott et al. 2015). Given that there is a large degree of ambiguity in the definition of polypharmacy, we sought to examine the level of agreement between clinicians when presented with a patient's medication list in their assessment of polypharmacy and to identify medication‐related characteristics that influence their assessment of polypharmacy.

## Materials and Methods

The study protocol was approved by the Human Research Ethics Committee of the Royal Adelaide Hospital (HREC/15/RAH/420).

### Clinical expert recruitment, distribution of questionnaire, and data collection

We invited national (Australian) clinical experts in the field of polypharmacy and deprescribing to participate in our study. Participants were identified based on their prior research in the subject matter, as well as those who have prior or active involvement in the Australian Deprescribing Network. Participant recruitment was done by email, utilizing an electronic survey software Qualtrics (Farrell and Raman‐Wilms 2015). Individual emails with links to the questionnaire were generated and sent to each expert inviting their participation. Completion of the questionnaire was participant consent.

### Case selection

Real life clinical cases were used to examine expert clinician assessment of polypharmacy. Medications and comorbid conditions of a total of 200 de‐identified patients who were seen as an outpatient in the Multidisciplinary Ambulatory Consultation Service (MACS) clinic at the Royal Adelaide Hospital (Ho et al. 2014) between 2014 and 2015 were independently reviewed by two investigators (SS and GO) to determine the number of comorbidities and medications for each case. Each patient in the MACS clinic is seen by a clinical pharmacist who conducts a comprehensive medication history with the patient with confirmation via a secondary source (e.g., GP list, pharmacy dispensing records). The criteria of assessment included;

1. Comorbid conditions: Only conditions, which are managed by regular pharmacological treatment, were considered. Conditions that require intermittent management such as migraines or infections, were excluded unless prior documentation of severe disease and hence requiring regular prophylaxis.
2. Medications: Only prescribed medications that are taken regularly were included. Over the counter medications such as herbal remedies were excluded, as were medications that are only used short term such as antibiotics courses or as required pain relief.

**Figure 1:**
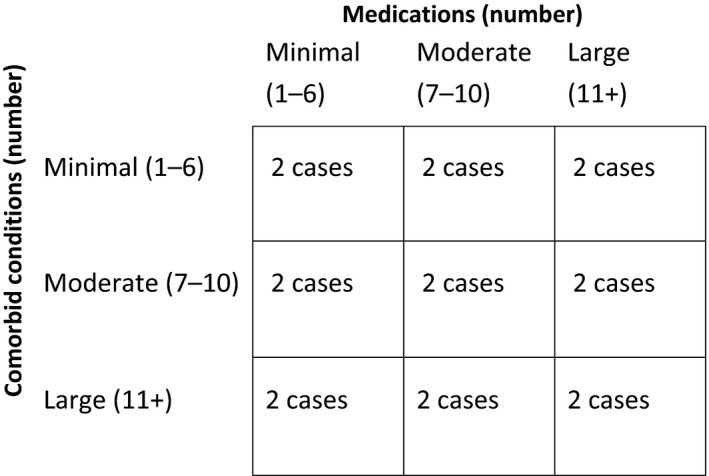
Case selection based on number of medications and comorbid conditions.

In order to select a range of cases with varying number of comorbidities and medications for expert clinical assessment, the total number of comorbid conditions and medications (as defined by the assessment criteria) were stratified into tertiles of low, medium, and large. Two cases from each tertile of comorbid conditions and medications were randomly selected using a random number generator for inclusion in the study (Fig. 1). Two control cases were also included; a positive control case for polypharmacy with a total of 25 prescribed medications and a negative control case with the patient taking one prescribed medication. A total of 20 de‐identified cases were selected to be included in our study.

### Determination of medication‐related factors influencing assessment of polypharmacy

For each of the 20 cases selected, the numbers of Beer's medicines(Campanelli 2012), STOPP criteria medicines(O'Mahony et al. 2015), falls‐risk medicines (Milos et al. 2014), Drug Burden Index (DBI) score (Hilmer et al. 2007), medicines causing postural hypotension, and potential pharmacokinetic drug–drug interactions (Flockhart 2007) were calculated independently by two authors (GO, SS). A summary of medication‐related characteristics of the cases included in the study is provided in Table 1.

**Table 1:** Overall medication‐related characteristics of patient cases included in study (*n* = 20)

Medication Characteristics	Scores
Total number of medicines (mean ± SD)	8.3 ± 4.1
Beers criteria medicines (mean ± SD)	1.0 ± 1.2
STOPP criteria medicines, median (IQR)	0.5 (IQR 0–3.3)
Falls‐risk medicines, median (IQR)	0 (IQR 0–1)
Drug burden index score, median (IQR)	0.5 (IQR 0–0.6)
Medicines causing postural hypotension, (mean ± SD)	1.6 ± 1.2
Pharmacokinetic drug–drug interactions, median (IQR)	3.5 (IQR 2‐8)

### Design of questionnaire

Each of the 20 cases were provided a common stem of “This is a 67‐year‐old patient living independently in the community”, in order to standardize the assessment of polypharmacy independent of a patient's age or functional state.

Each case was given the same set of questions:

1. How would you rate this patient's degree of polypharmacy from no polypharmacy (0) to extreme polypharmacy (10)?
2. How would you rate this patient's medications based on their ability to be associated with harm from no risk of harm (0) to almost certain harm (10)?
3. How would you rate this patient's medications based on potential to have the medication list reduced or simplified assuming that patient is compliant and that you have infinite resources, from no potential to be reduced or simplified (0) to maximum potential (10)?

Both the stem and set of questions were piloted with the case scenarios by the research team with three separate groups of other experts in polypharmacy, in iterative steps to ensure face and content validity.

### Statistical analysis

Inter‐rater reliability analysis between expert clinical participants for each of the three questions was conducted to examine the intraclass correlation coefficients (ICC) for each case. Inter‐rater reliability was interpreted as slight (0.00–0.20), fair (0.21–0.40), moderate (0.41–0.60), substantial (0.61–0.80), and almost perfect (0.81–1.00).(Landis and Koch 1977).

Shapiro–Wilk test of normality was used to examine if the data for each of the variables were normally distributed. Correlation analyses between each of the three questions in the survey were conducted, using Pearson's for normally distributed data and Spearman for non‐normally distributed data.

To identify medicine factors predictive of polypharmacy scores, medications likely to cause harm, and ability to deprescribe, univariate linear regression was conducted. Those variables with a *P* ≤ 0.10 in the univariate analyses were then included in the backward stepwise multivariate logistic regression analyses, with a significance level of *P* < 0.05 assigned for inclusion in the final model. *R* ^2^ value was used to assess the goodness‐of‐fit of the final models.

All statistical analyses were performed using SPSS Version 22 (SPSS Inc, Chicago, IL).

## Results

### Participant and case characteristics

Responses were received from a total of 18 expert clinicians from 26 invitations sent (69.2% response rate). Nine of the participants were clinical pharmacists and nine were medical practitioners, including general physicians, geriatricians, and clinical pharmacologists.

### Agreement between expert clinicians

There was strong agreement between each of the expert clinicians included in the study and their assessment of polypharmacy (ICC = 0.94), likelihood to cause harm (ICC = 0.89), and ability to simplify the medication list (ICC = 0.90).

### Correlations of assessment of cases with individual medication‐related factors

There were significant correlations between clinicians' polypharmacy scores given to the cases and the total number of medications (*R* = 0.92, *P* < 0.0001), presence of falls‐risk medications (*R* = 0.54, *P* = 0.02), and their DBI scores (*R* = 0.57, *P* = 0.01) (Table 2). Assessment of the likelihood to cause harm in the cases presented was significantly correlated with total numbers of medicines (*R* = 0.68, *P* = 0.002), Beers criteria medicines (*R* = 0.69, *P* = 0.001), falls‐risk medicines (*R* = 0.67, *P* = 0.002), DBI scores (*R* 0.68, *P* = 0.002), and presence of pharmacokinetic drug interactions (*R* = 0.50, *P* = 0.04) (Table 2). For the assessment of ability to simplify the medications, all of the medication‐related factors were positively correlated aside from medicines causing postural hypotension and pharmacokinetic drug–drug interactions (Table 2).

**Table 2:** Correlations of assessment of cases with individual medication‐related factors

		Polypharmacy score	Likelihood to cause harm	Ability to simplify
Total number of medicines^a^	*R*	**0.92**	**0.68**	**0.67**
	*P* ‐value	**<0.0001**	**0.002**	**0.002**
Beers criteria medicines^a^	*R*	0.39	**0.69**	**0.50**
	*P* ‐value	0.11	**0.001**	**0.03**
STOPP criteria medicines	*R*	0.45	0.34	**0.59**
	*P* ‐value	0.06	0.17	**0.01**
Falls‐risk medicines	*R*	**0.54**	**0.67**	**0.58**
	*P* ‐value	**0.02**	**0.002**	**0.01**
Drug burden index	*R*	**0.57**	**0.68**	**0.57**
	*P* ‐value	**0.01**	**0.002**	**0.01**
Medicines causing postural hypotension	*R*	0.42	0.28	0.16
	*P* ‐value	0.09	0.27	0.54
Pharmacokinetic drug–drug interactions	*R*	0.26	**0.50**	0.04
	*P* ‐value	0.29	**0.04**	0.87

a Normally distributed data were examined using Pearson's correlation coefficient, and all other variables were examined using Spearman's correlation coefficient. Values highlighted in bold are statistically significant.

### Medication‐related factors predictive of expert clinical assessment scores

Multivariate analyses demonstrated that the number of medicines (*P* < 0.0001) and the DBI scores (*P* = 0.047) were significantly associated with the assessment of the level of polypharmacy by the clinical experts (Table 3). Clinical expert assessments of medicines associated with harm were significantly associated with the number of medicines (*P* = 0.01) and Beers criteria medicines (*P* = 0.003). Ability to simplify the medication regimen was significantly associated with number of medicines (*P* = 0.03) and medicines from the STOPP criteria (*P* = 0.018) (Table 3).

**Table 3:** Stepwise multivariate analyses of medication‐related factors predictive of polypharmacy score, likelihood to cause harm, or ability to simplify

Medication Factor	*R* ^2^	Beta (coefficient)	*t*	*P* ‐value
Polypharmacy score	0.89			
Number of medicines		0.83	8.45	<0.0001
Drug burden index		0.21	2.17	0.047
Medicines associated with harm	0.73			
Number of medicines		0.57	4.01	0.01
Beers medicines		0.49	3.5	0.003
Ability to simplify medicines	0.64			
Number of medicines		0.44	2.41	0.03
STOPP criteria		0.48	2.65	0.018

Variables from the univariate analyses with a *P* < 0.10 were included in the multivariate stepwise linear regression model and falls‐risk medicine was excluded from the model to avoid collinearity with Drug Burden Index (DBI).

*R* ^2^ assesses goodness‐of‐fit of model.

## Discussion

To the best of our knowledge, this is the first study to explore quantitatively, how clinicians assess polypharmacy and the influence of medication‐related factors on their assessment. Despite the varying existing definitions of polypharmacy, and the fact that we did not provide any guidance or definition for polypharmacy in our survey or invitation, we have shown that there is a very strong consensus between expert clinicians with regard to assessment of polypharmacy, medication harm, and ability to simplify medications. Of the medication‐related factors examined, the number of medicines and the DBI scores were positively associated with assessment of the level of polypharmacy. Number of medicines was also associated with clinician assessment of medicines that are likely to cause harm and ability to simplify medication regimen, as was the presence of Beers Criteria and STOPP medicines, respectively.

While there was a strong association between number of medicines prescribed to patients and assessment of polypharmacy, this study has shown that the presence of medicines commonly associated with harm particularly in the older population (DBI, Beers and STOPP medicines), significantly influences clinicians assessment of appropriateness of medication regimens. It should be highlighted that these assessments were made without the provision of these lists/tools by the research team.

Both the Beers and STOPP Criteria are designed to assist health care professionals in identification of potentially inappropriate medication use in older adults (Campanelli 2012; O'Mahony et al. 2015). A number of studies have found that the use of STOPP was more likely to identify serious adverse drug events in hospitalized patients, a wider range of medication‐related problems and is a more effective tools for reducing inappropriate medication use, by comparison to the Beers criteria (Curtain et al. 2013). In this study, Beers criteria medicines were associated with clinicians' assessment of inappropriate medicines most likely to be associated with harm and STOPP medicines most likely to be minimize medication regimens, reflecting the specific focus and utilization of these prescribing tools.

A strength of this study is the utilization of de‐identified “real‐world” cases and the random selection of cases, with an even distribution of the number of medications and comorbidities, ensuring applicability of the assessments to current practice. However, there are limitations to our study. The number of cases clinicians assessed was chosen to maximize variability of the cases, with the use of stratified random selection, while minimizing the potential for respondent fatigue, and is possible that participants did not receive a full scope of all possible numbers of medications and specific medication combinations. Secondly, we also excluded all over‐the‐counter medications that were not prescribed regularly by their doctors. Over‐the‐counter medications can be an important contributor to the problem of polypharmacy. Approximately one‐third of older Australians take at least one over‐the‐counter or complementary medication and these medications can cause harm and may contribute to potentially unknown drug–drug and drug‐disease interactions (Goh et al. 2009).

In summary, this study demonstrates that despite varying definitions of polypharmacy in the literature, when a group of clinical experts in polypharmacy assess medication lists there was a high degree of agreement on scoring of polypharmacy, as well as the assessment of risks of harm and potential for deprescribing. We have also shown that the perception of polypharmacy correlates with several aspects of medication use; the strongest correlation was with the number of medications used, but was also associated with various measures of the potential for the medicine to cause harm, and to be inappropriate. Future research on definitions of polypharmacy should take these factors into account, and validate them against clinical outcomes.

## Disclosure

All authors have completed the Unified Competing Interest form at http://www.icmje.org/coi_disclosure.pdf (http://www.icmje.org/coi_disclosure.pdf) (available on request from the corresponding author) and declare no support from any organization for the submitted work.
